# Combined in-office endoscopic debridement and supine head-hanging budesonide instillation for postoperative difficult-to-treat chronic rhinosinusitis with nasal polyps: a retrospective cohort study

**DOI:** 10.3389/falgy.2026.1914838

**Published:** 2026-08-25

**Authors:** Lili Wang, Minghui Zhou, Shanpeng Yuan, Yang Jiao, Luping Wang, Yunpeng Ba, Qingwen Zhu

**Affiliations:** Department of Otolaryngology-Head and Neck Surgery, The First Affiliated Hospital of Zhengzhou University, Zhengzhou, China

**Keywords:** allergic rhinitis, chronic rhinosinusitis with nasal polyps, difficult-to-treat chronic rhinosinusitis, endoscopic debridement, mucosal epithelialization, supine head-hanging budesonide instillation

## Abstract

**Background:**

Difficult-to-treat chronic rhinosinusitis with nasal polyps (CRSwNP) may remain uncontrolled after adequate endoscopic sinus surgery and guideline-directed postoperative therapy. Obstructive edematous or polypoid tissue in the operated sinus cavity may limit topical corticosteroid access and delay mucosal recovery. We evaluated whether an intensified outpatient strategy—targeted in-office endoscopic debridement followed by supine head-hanging budesonide instillation—was associated with earlier recorded postoperative cavity epithelialization.

**Methods:**

This single-center retrospective cohort included 102 adults treated from January 2020 to January 2025 and reassessed against EPOS 2020 difficult-to-treat criteria. Fifty-two patients received the intensified strategy, and 50 received conventional budesonide nasal spray; both groups received the same 7-day oral methylprednisolone course. The primary endpoint was recorded postoperative cavity epithelialization at 12 weeks. Secondary endpoints included 8-week epithelialization, time to first recorded epithelialization, symptom and Lund-Kennedy score improvement, exploratory adjusted association with 12-week epithelialization, and adverse events documented in records. Because the intensified strategy comprised multiple co-delivered components, the study evaluated the overall strategy, not individual components.

**Results:**

Epithelialization by week 8 was recorded in 43/52 (82.69%) intensified-strategy patients and 20/50 (40.00%) conventional-strategy patients (*P* < 0.001). Twelve-week epithelialization was recorded in 46/52 (88.46%) and 32/50 (64.00%) patients, respectively (risk difference, 24.46 percentage points; 95% CI, 8.57–40.35; risk ratio, 1.38; 95% CI, 1.10–1.74; *P* = 0.004). Median time to first recorded epithelialization was 4 versus 12 weeks (log-rank *P* < 0.001). Holm-adjusted comparisons favored the intensified strategy for symptom and endoscopic score improvements. In exploratory Firth regression, the intensified strategy remained associated with 12-week epithelialization (adjusted OR, 5.58; 95% CI, 1.87–16.68; *P* = 0.002). Serious adverse events were not documented, but safety assessment was retrospective and incomplete.

**Conclusion:**

In adults with postoperative difficult-to-treat CRSwNP, the intensified outpatient strategy was associated with earlier and more frequent recorded postoperative cavity epithelialization than conventional budesonide nasal spray. These findings indicate a strategy-level association and do not isolate component effects or establish comparative safety. The 12-week endpoint reflects early recorded epithelialization and does not establish durable disease control or recurrence prevention. Prospective component-controlled or factorial studies with follow-up of at least 12 months are needed.

## 1 Introduction

Chronic rhinosinusitis with nasal polyps (CRSwNP) is a chronic inflammatory disorder characterized by persistent sinonasal symptoms and objective evidence of bilateral nasal polyps or sinonasal mucosal disease. Current consensus documents emphasize that diagnosis, assessment of disease control, and treatment selection should integrate both patient-reported symptoms and objective findings (1–3). CRSwNP can substantially impair nasal breathing, olfaction, sleep, daily functioning, and work productivity, while repeated medical treatment and surgery impose a considerable burden on patients and health-care systems (4). Although CRSwNP is clinically and biologically heterogeneous, postoperative management commonly depends on controlling persistent mucosal inflammation, promoting mucosal recovery, and maintaining access for topical therapy (5–8).

Allergic rhinitis (AR) is a common comorbidity in chronic inflammatory upper-airway disease and may aggravate nasal obstruction, rhinorrhea, and mucosal edema in patients with postoperative CRSwNP. AR and CRSwNP are distinct entities, but overlapping type 2 inflammatory features may complicate symptom interpretation and postoperative disease assessment (9–11). In the present study, AR was considered a clinically relevant comorbidity and potential effect modifier, rather than a presumed cause of postoperative recurrent polypoid inflammation.

Endoscopic sinus surgery (ESS) improves sinonasal ventilation, drainage, and access for topical therapy, but it does not eliminate the underlying inflammatory tendency. A subset of patients continues to have persistent symptoms and endoscopic disease despite technically adequate surgery and postoperative medical treatment. EPOS 2020 defines difficult-to-treat rhinosinusitis as persistent disease despite appropriate surgery and intranasal corticosteroid therapy, with allowance for up to two short courses of antibiotics or systemic corticosteroids in the preceding year (1). In the present study, delayed epithelialization, mucosal edema, or recurrent polypoid changes alone were not used as independent eligibility criteria for difficult-to-treat CRSwNP; these findings were interpreted as postoperative endoscopic evidence of persistent disease activity only after patients had fulfilled the EPOS 2020 difficult-to-treat framework.

Topical corticosteroids remain central to postoperative CRSwNP management. Their clinical effect depends not only on the anti-inflammatory activity of the corticosteroid, but also on whether the medication can reach the operated sinus cavity. Delivery device, administered dose, solution volume, head position, treatment duration, postoperative anatomy, and patient adherence may all influence local drug distribution. Randomized studies and pooled analyses support large-volume or otherwise enhanced corticosteroid delivery after ESS for improving symptom and endoscopic outcomes, although protocols differ in dose, volume, frequency, device, position, and duration and should not be considered interchangeable (12–23).

Routine-care evidence remains limited on how enhanced topical corticosteroid delivery should be integrated with outpatient management of obstructive inflammatory tissue within the operated sinus cavity. Edematous, polypoid, granulation, or fibrinous tissue may narrow an otherwise adequate surgical opening and reduce mucosal exposure to topical medication. Conventional nasal sprays deliver a relatively small volume, whereas irrigation or direct instillation may reach a larger postoperative mucosal surface. Few routine-care studies have evaluated a combined outpatient strategy that first reduces obstructive inflammatory tissue and then applies gravity-dependent corticosteroid delivery in an EPOS-defined difficult-to-treat adult cohort.

The combined intensified outpatient strategy examined in this study was designed to address two potentially modifiable postoperative barriers: obstructive inflammatory tissue within the operated sinus cavity and limited access of topical medication. Targeted in-office endoscopic debridement was intended to reduce the obstructive inflammatory burden while preserving the sinus mucosa as far as possible, and supine head-hanging budesonide instillation was then used to increase contact between the medication and the postoperative mucosal surface. Because both groups received the same initial 7-day course of oral methylprednisolone, the comparison focused on different postoperative local-treatment strategies rather than differential systemic corticosteroid use. We therefore evaluated whether the combined intensified strategy was associated with earlier and more frequent recorded postoperative cavity epithelialization over 12 weeks.

## 2 Materials and methods

### 2.1 Study design and reporting

We conducted a single-center retrospective cohort study at the Department of Otolaryngology-Head and Neck Surgery, The First Affiliated Hospital of Zhengzhou University, Zhengzhou, China. Medical records from January 2020 through January 2025 were reviewed. The index date was the documented initiation of the postoperative strategy evaluated in this study, and outcomes were assessed through 12 weeks. The analysis compared two management strategies used in routine clinical care among adults with EPOS 2020-defined difficult-to-treat CRSwNP. Treatment allocation was not randomized, and no research-mandated allocation protocol determined the choice of strategy. The study did not alter clinical care. Reporting followed the Strengthening the Reporting of Observational Studies in Epidemiology statement (24).

The Ethics Committee of The First Affiliated Hospital of Zhengzhou University approved the study (2025-KY-2115-002). The study was conducted in accordance with the Declaration of Helsinki. All patients provided written informed consent for clinical treatment and follow-up. The committee waived additional consent for this retrospective analysis of de-identified clinical data.

### 2.2 Participants

Before analysis, source records and the aggregate screening log were re-reviewed against the EPOS 2020 difficult-to-treat criteria. At the index visit, all participants were aged 18 years or older and had CRSwNP with persistent symptoms and endoscopic disease despite appropriate treatment. Prior management included adequate ESS, continued intranasal corticosteroid treatment, and up to two short courses of antibiotics or systemic corticosteroids during the preceding year. The aggregate screening log identified 150 postoperative CRSwNP records screened for eligibility. During re-audit, exclusion criteria were applied sequentially in the fixed hierarchical order shown in Supplementary Table S1, and each record was counted only once under the first criterion met in that sequence. The resulting reason-specific counts were mutually exclusive and summed to 48: age younger than 18 years, *n* = 3; inability to safely adopt the supine head-hanging position, *n* = 2; medical contraindications, *n* = 8; specific non-CRSwNP etiologies, *n* = 2; pregnancy or lactation, *n* = 1; incomplete records or an indeterminate 12-week endpoint, *n* = 3; and initiation of additional potentially confounding treatments during follow-up, *n* = 29. The restricted final analytic cohort included 102 eligible adults with complete treatment allocation, baseline analysis variables, and recorded postoperative cavity epithelialization status through week 12.

Persistent non-epithelialized inflammation or recurrent postoperative polypoid disease was accepted as evidence of ongoing endoscopic disease only after the treatment-history requirements had been reviewed. These findings were not treated as substitutes for adequate prior surgery or medication exposure. The analytic cohort therefore represents postoperative difficult-to-treat disease rather than an unselected group with any persistent or recurrent postoperative abnormality.

We excluded patients younger than 18 years and those with cervical spine disease or other conditions precluding safe use of the supine head-hanging position. Medical exclusions included severe cardiovascular or cerebrovascular disease, uncontrolled hypertension or diabetes, active peptic ulcer disease, severe osteoporosis, and glaucoma. Serious hepatic, renal, or hematologic disease and hypersensitivity to the study medications were also exclusion criteria. We further excluded cystic fibrosis, primary ciliary dyskinesia, fungal sinusitis, pregnancy or lactation, incomplete records, and an indeterminate 12-week endpoint. These criteria reflected diagnostic specificity and the safety considerations relevant to positioning and corticosteroid exposure.

### 2.3 Treatment strategies

Patients were grouped according to the postoperative treatment strategy documented and initiated in routine clinical records. The intensified strategy consisted of targeted in-office endoscopic debridement followed by supine head-hanging budesonide instillation; both groups received the same initial short course of oral methylprednisolone, as detailed below. In routine care, selection of the intensified strategy was based on the treating rhinologist's judgment and shared clinician-patient decision-making, and may have been influenced by postoperative endoscopic appearance, accessibility of the operated sinus cavity, the presence of obstructive edematous or polypoid inflammatory tissue, patient tolerance of the supine head-hanging position, prior response to topical therapy, follow-up feasibility, and patient preference. After adequate topical nasal anesthesia, markedly edematous, vesicle-like, or polypoid tissue within the opened sinus cavities was removed under endoscopic guidance using mucosal forceps, biting forceps, or a microdebrider when necessary. Debridement was limited to obstructive inflammatory tissue within the opened sinus cavity; the procedural objective was to restore visualization, drainage, and access for topical therapy while preserving intact mucosa as far as possible. When needed, cotton pledgets containing local anesthetic and a small amount of epinephrine were applied temporarily for hemostasis, and routine packing of the sinus cavity was not performed. This procedure represented a limited, office-based, endoscopically guided debridement of obstructive inflammatory tissue and did not constitute formal revision endoscopic sinus surgery.

After debridement, patients in the intensified group assumed the supine head-hanging position for budesonide instillation (Figure 2a). Budesonide suspension was supplied at 1 mg/2 mL. A 1-mL dose was instilled into each nasal cavity twice daily (morning and evening), for a total daily dose of 2 mg. Patients maintained the position for 10–20 min after instillation. Treatment continued until recorded postoperative cavity epithelialization or week 12, whichever occurred first. The conventional group used budesonide nasal spray (64 μg per actuation), one actuation per nostril twice daily, for a total daily dose of 256 μg. Both groups received oral methylprednisolone 24 mg once each morning for 7 days. Both groups underwent scheduled endoscopy every 2 weeks during the 12-week observation period. In the intensified group, targeted debridement was performed at the index visit and repeated at each 2-week visit while the postoperative cavity remained non-epithelialized. Debridement stopped after epithelialization was recorded or at the end of week 12. Each session lasted approximately 10–15 min from the start of topical nasal anesthesia to completion; duration varied with the extent and location of obstructive inflammatory tissue. All procedures were completed during a single outpatient visit without additional anesthesia or sedation. No patient initiated biologic therapy during the observation period.

Thus, the groups received different local-treatment packages against the same short course of systemic corticosteroid. The intensified strategy differed in procedural exposure, budesonide formulation, nominal topical dose, delivery position, and possible repeat debridement. Because these components were co-delivered, the analysis estimates the association between the overall intensified strategy and outcomes. It cannot identify the independent contribution of debridement, budesonide instillation, topical dose, delivery position, or repeat-intervention frequency. The comparison was not dose-equivalent and should not be interpreted as isolating a pharmacologic or positional effect.

### 2.4 Data collection and follow-up

Demographic, inflammatory, symptom, endoscopic, treatment, and outcome variables were abstracted from de-identified clinical records. Demographic and inflammatory variables comprised age, sex, peripheral blood eosinophil percentage, serum total IgE, and tissue eosinophil count per high-power field. Clinical variables comprised three symptom VAS measures and the Lund-Kennedy score. Follow-up endoscopy, symptom scores, rescue treatment, and treatment escalation were reviewed through week 12 when available. Additional clinically relevant baseline and historical domains included asthma diagnosis and control, confirmed allergic rhinitis and allergen sensitization, prior ESS burden and revision surgery, prior biologic exposure, medication adherence, concomitant anti-allergic or asthma-related medication use, and repeat-debridement frequency or timing. However, routine records did not provide these domains as sufficiently standardized, analysis-ready patient-level variables for reproducible cohort-level abstraction. Variable-specific availability and missingness percentages could therefore not be reported without retrospective reconstruction. These domains were not imputed, used for effect-modification analysis, or included in multivariable adjustment. During the 12-week observation period, endoscopic examinations were performed under standardized outpatient conditions by the same rhinology team. A 30° rigid nasal endoscope was used, and the operated cavities were inspected systematically in a fixed sequence, including the maxillary, ethmoid, sphenoid, and frontal sinus regions when surgically opened. Endoscopic images were stored at each visit whenever available and were used for endpoint verification during data abstraction. Patients received standardized instructions for twice-daily budesonide instillation in the supine head-hanging position. Actual adherence to the prescribed position, positioning duration, and instillation frequency was not captured using a structured diary, electronic monitor, or other objective measure; treatment fidelity could therefore not be quantified.

### 2.5 Outcomes

The primary endpoint was postoperative cavity epithelialization recorded by week 12, derived directly from the binary outcome-status field in the source dataset. This status field was assigned contemporaneously in routine care by the treating rhinology team on the basis of postoperative endoscopic assessment. Status 1 denoted that postoperative cavity epithelialization had been recorded, whereas status 0 denoted that epithelialization had not been recorded by week 12. The source status field was the analysis variable. It represented a single binary endoscopic endpoint, not a composite endpoint. Routine descriptive findings, including mucosal color or smoothness, exposed bone, fibrin, granulation, edema, polypoid change, or drainage-pathway patency, could inform the treating team's endoscopic judgment but were not separately coded, combined, or required as independent endpoint components. Antibiotic use, rescue systemic corticosteroid use, additional procedures or revision surgery, and initiation or escalation of biologic therapy were not endpoint components.

Endpoint generation and retrospective verification were distinct steps. The binary status was first recorded during routine, unblinded clinical care. During data abstraction, two experienced rhinologists independently checked whether the source status was supported by the contemporaneous endoscopic documentation and stored images when available. Discrepancies or uncertainty were resolved by consensus, with involvement of a third rhinologist when necessary, before the analysis dataset was locked. This process verified the source-coded binary status; it did not reconstruct a composite endpoint from separately coded endoscopic features. Before formal verification, the assessors jointly reviewed postoperative endoscopic images from 20 non-study patients to harmonize their interpretation. Because the initial independent verification ratings were not retained as a structured dataset, formal interobserver-agreement statistics could not be calculated. Blinded central adjudication was not performed.

Secondary endpoints were 8-week recorded postoperative cavity epithelialization, time to first recorded postoperative cavity epithelialization, nasal obstruction VAS score, rhinorrhea VAS score, head fullness VAS score, Lund-Kennedy endoscopic score, exploratory adjusted association with 12-week recorded postoperative cavity epithelialization, and adverse events documented in the clinical records. VAS scores ranged from 0 to 10, with higher scores indicating more severe symptoms. Higher Lund-Kennedy scores indicated more severe endoscopic disease. Recorded adverse events included epistaxis requiring intervention, vasovagal reaction, severe pain, acute infection, intolerance of the supine head-hanging position, and systemic corticosteroid-related complications such as uncontrolled hypertension or hyperglycemia.

### 2.6 Statistical analysis

All eligible records available for the prespecified study period were analyzed. No *a priori* sample-size calculation was performed because of the retrospective design; estimate precision was characterized using 95% confidence intervals. Approximately symmetric continuous variables were summarized as mean ± standard deviation and compared with Welch's *t* test. Ordinal or non-normally distributed variables were summarized as median (interquartile range) and compared with the Mann–Whitney U test. Categorical variables were reported as *n* (%) and compared with Pearson's chi-square test or Fisher's exact test, as appropriate. For continuous and ordinal variables, standardized mean differences (SMDs) were calculated as the difference in group means divided by the pooled standard deviation; for sex, the standardized difference in proportions was used. SMDs were descriptive measures of group imbalance and were not used to claim baseline equivalence. All analysis variables were complete.

For binary outcomes, absolute risk differences with Wald 95% confidence intervals were reported, together with risk ratios and crude odds ratios with log-scale 95% confidence intervals. The 12-week epithelialization endpoint was considered the primary outcome, whereas all other outcomes were interpreted as secondary or supportive. Between-group comparisons of the four score-improvement outcomes were performed using Mann–Whitney U tests, with Holm correction applied to control the family-wise error rate within this outcome family. Rank-biserial correlation was used to quantify the corresponding effect size. Time to first recorded epithelialization was defined as the interval from treatment initiation to the first scheduled or clinically indicated endoscopic visit at which epithelialization was documented. Groups were compared using Kaplan–Meier curves and the log-rank test. Patients without recorded epithelialization by week 12 were administratively censored at 12 weeks. The event time was therefore interpreted as the first documented visit at which epithelialization was observed, rather than the exact biological onset of epithelialization. Because visits occurred at scheduled follow-up windows, recorded epithelialization may have occurred later than the true biological onset of mucosal repair.

Because the number of patients without recorded 12-week epithelialization was limited, multivariable analysis was performed using a parsimonious Firth bias-reduced logistic regression model to reduce small-sample separation bias. The model included treatment strategy, age, sex, peripheral blood eosinophil percentage, and baseline Lund-Kennedy score. Covariate selection was constrained by the limited number of non-epithelialization events and by the availability of consistently recorded variables. Important potential confounders, including confirmed allergic rhinitis, allergen sensitization, asthma diagnosis and control, prior number of ESS procedures, previous revision surgery, medication adherence, concomitant anti-allergic or asthma-related medication use, prior biologic exposure, and detailed postoperative inflammatory burden, were not sufficiently structured or complete for inclusion. These variables were not imputed because their absence reflected inconsistent routine-care documentation rather than isolated missing cells. Residual confounding could therefore not be excluded. Adjusted odds ratios (ORs), Wald 95% confidence intervals (CIs), and two-sided *P* values were reported. This exploratory model was neither a prediction model nor evidence that the treatment association was independent of unmeasured confounding. Statistical significance was defined as *P* < 0.05. Analyses were performed using Python 3.12; the Firth model was fitted with a custom implementation of bias-reduced logistic regression.

## 3 Results

### 3.1 Cohort and baseline characteristics

**Figure 1:**
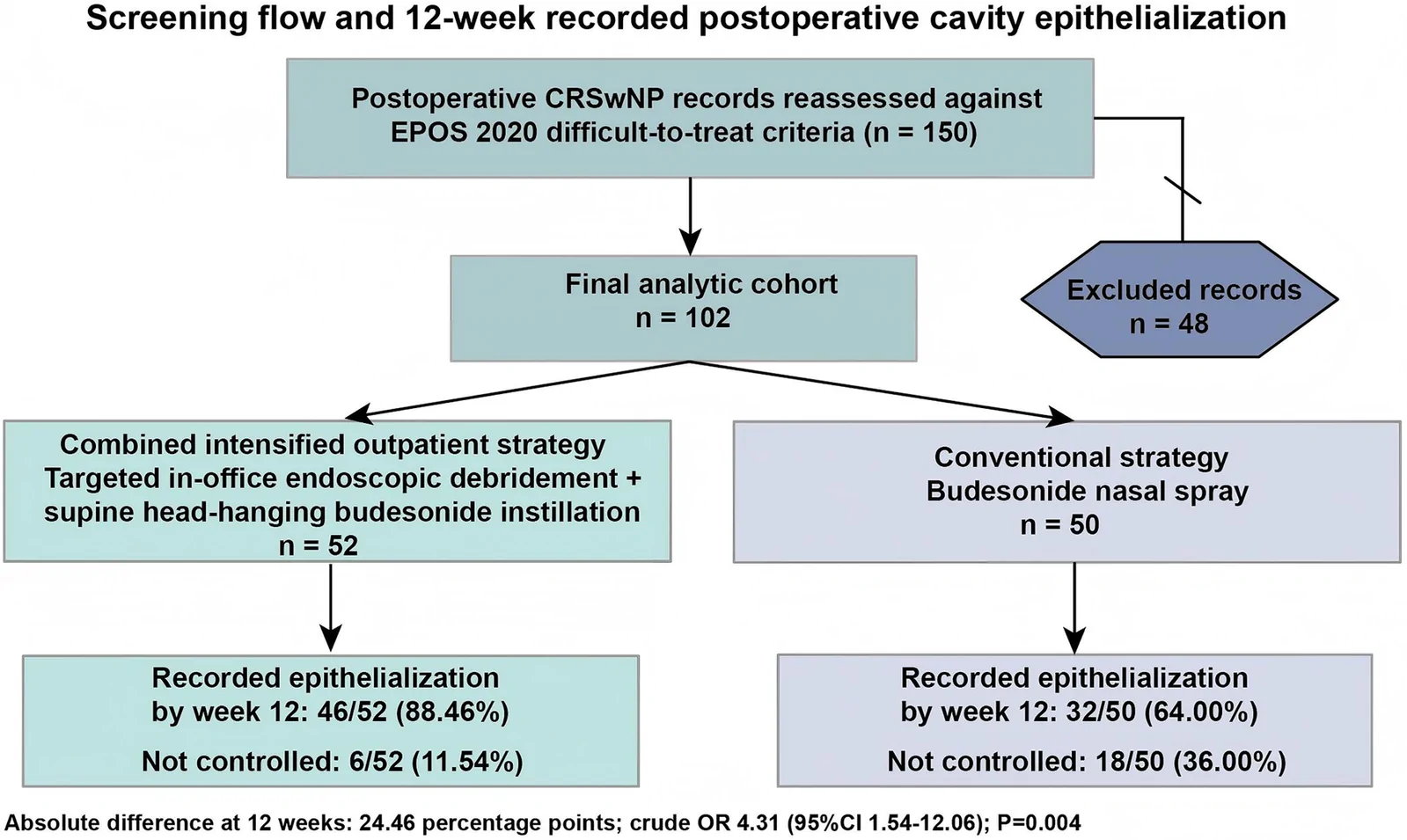
Screening flow and postoperative cavity epithelialization recorded by week 12. A total of 150 postoperative CRSwNP records were screened against the EPOS 2020 difficult-to-treat framework. Exclusion criteria were applied sequentially in the fixed hierarchical order shown in Supplementary Table S1, with each record counted once under the first criterion met. The intensified and conventional groups included 52 and 50 patients, respectively. Postoperative cavity epithelialization was recorded by week 12 in 46/52 (88.46%) and 32/50 (64.00%) patients, respectively. Post-index exclusion of patients who initiated additional potentially confounding treatment remains a potential source of selection bias.

Of 150 screened postoperative CRSwNP records, 48 were excluded, and 102 eligible adults with complete treatment allocation, baseline data, and outcome data were included in the restricted final analytic cohort. The detailed screening process is shown in Figure 1. Among the included patients, 52 received the combined intensified outpatient strategy and 50 received the conventional strategy. Age ranged from 18.5 to 70.0 years. Mean age was 39.96 ± 13.73 years in the intensified group and 39.37 ± 12.61 years in the conventional group (*P* = 0.822). Men accounted for 29/52 (55.8%) and 35/50 (70.0%) patients, respectively (*P* = 0.137). The largest baseline imbalance was observed for sex (SMD = 0.295), which was retained in the exploratory adjusted model. Absolute SMDs for all other reported characteristics ranged from 0.045 to 0.205 (Table 1).

**Table 1:** Baseline demographic, inflammatory, symptom, and endoscopic characteristics.

Characteristic	Intensified (*n* = 52)	Conventional (*n* = 50)	SMD	*P* value
Age, years	39.96 ± 13.73	39.37 ± 12.61	0.045	0.822
Male sex, *n* (%)	29 (55.8)	35 (70.0)	−0.295	0.137
Peripheral blood eosinophils, %	7.39 ± 1.79	7.74 ± 1.65	−0.205	0.302
Serum total IgE, IU/mL	178.88 ± 58.22	172.36 ± 61.74	0.109	0.584
Tissue eosinophils, cells/HPF	49.48 ± 15.55	50.38 ± 15.70	−0.058	0.772
Nasal obstruction VAS	7.00 (5.00–8.00)	7.00 (5.00–8.00)	0.089	0.600
Rhinorrhea VAS	8.00 (6.75–8.25)	8.00 (7.00–9.00)	−0.104	0.538
Head fullness VAS	4.00 (3.00–5.00)	4.00 (2.00–5.00)	0.184	0.518
Lund-Kennedy score	5.00 (4.00–5.00)	5.00 (4.00–5.00)	0.086	0.638

Approximately symmetric continuous variables are presented as mean ± SD, ordinal symptom and endoscopic scores as median (IQR), and sex as *n* (%). *P* values were calculated with Welch's *t* test, the Mann–Whitney U test, or Pearson's chi-square test, as appropriate. Continuous and ordinal SMDs were calculated as the difference in group means divided by the pooled SD; the standardized difference in proportions was used for sex. Baseline *P* values are descriptive and do not demonstrate equivalence. HPF, high-power field; IQR, interquartile range; SD, standard deviation; SMD, standardized mean difference; VAS, visual analog scale. Asthma status and control, confirmed allergic rhinitis or allergen sensitization, prior ESS burden including previous revision surgery, medication adherence, concomitant anti-allergic or asthma-related treatment, and previous biologic exposure were not sufficiently standardized as analysis-ready patient-level variables for reproducible cohort-level abstraction. Variable-specific availability and missingness percentages could not be reported without retrospective reconstruction; these domains were therefore not included as formal baseline characteristics, effect modifiers, or multivariable covariates.

Peripheral blood eosinophil percentage, serum total IgE, and tissue eosinophil counts were similar in descriptive comparisons. Baseline medians were also comparable for nasal obstruction VAS, rhinorrhea VAS, head fullness VAS, and Lund-Kennedy score. All baseline *P* values exceeded 0.13, but these tests were interpreted descriptively because nonsignificance does not demonstrate equivalence in a nonrandomized cohort.

Asthma diagnosis and control, confirmed allergic rhinitis or allergen sensitization, prior ESS burden including previous revision surgery, medication adherence, concomitant anti-allergic or asthma-related treatment, and prior biologic exposure were not included in Table 1 because routine records did not provide sufficiently standardized, analysis-ready variables for reproducible cohort-level abstraction. Variable-specific availability and missingness percentages were therefore unavailable without retrospective reconstruction, and these domains were not used in effect-modification analyses or multivariable adjustment.

**Figure 2:**
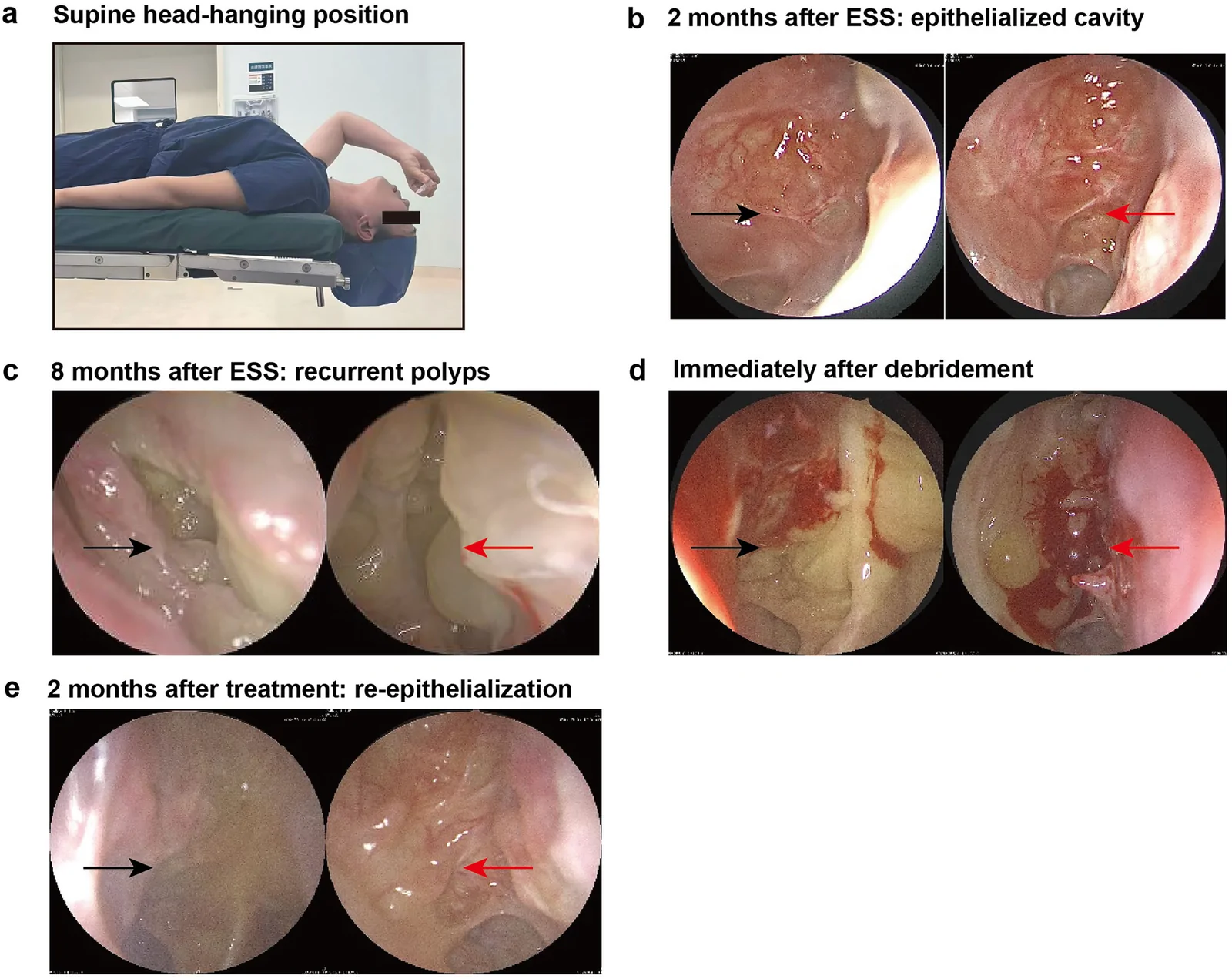
Supine head-hanging budesonide instillation and serial endoscopic findings in a representative patient with postoperative recurrent CRSwNP. **(a)** Supine head-hanging position used for budesonide suspension instillation. **(b)** Complete epithelialization of the postoperative sinus cavities 2 months after endoscopic sinus surgery. **(c)** Bilateral recurrent nasal polyps 8 months after endoscopic sinus surgery. **(d)** Immediate endoscopic appearance after in-office removal of polypoid tissue under local anesthesia. **(e)** Re-epithelialization of the postoperative sinus cavities 2 months after targeted debridement combined with positional budesonide instillation. Black arrows indicate the left ethmoid and sphenoid sinus regions, and red arrows indicate the right ethmoid and sphenoid sinus regions. Panels b–e show serial endoscopic findings from the same representative patient, illustrating the transition from postoperative epithelialization to recurrent polypoid disease, immediate post-debridement appearance, and subsequent re-epithelialization after the intensified outpatient strategy. All images were de-identified. The images illustrate the clinical sequence and do not independently establish treatment effect. CRSwNP, chronic rhinosinusitis with nasal polyps; ESS, endoscopic sinus surgery.

Figure 2 shows the supine head-hanging treatment position and a representative clinical sequence. In the endoscopic panels, black arrows indicate the left ethmoid and sphenoid sinus regions, whereas red arrows indicate the right ethmoid and sphenoid sinus regions. The serial images show postoperative cavity epithelialization 2 months after ESS, bilateral recurrent nasal polyps 8 months after ESS, the immediate appearance after targeted endoscopic debridement, and re-epithelialization 2 months after the combined intensified outpatient strategy. The figure illustrates the procedure and observed sequence but does not independently establish a treatment effect.

### 3.2 Primary endpoint at 12 weeks

At 12 weeks, recorded epithelialization was observed in 46/52 patients in the intensified-strategy group and 32/50 in the conventional-strategy group. The corresponding proportions were 88.46% and 64.00% (*χ*^2^ = 8.477; *P* = 0.004). Six (11.54%) and 18 (36.00%) patients, respectively, had no recorded epithelialization by week 12. The risk difference was 24.46 percentage points (95% CI, 8.57–40.35), and the risk ratio was 1.38 (95% CI, 1.10–1.74). The crude OR was 4.31 (95% CI, 1.54–12.06; Table 2).

**Table 2:** Binary epithelialization and time-to-recorded-epithelialization outcomes.

Outcome	Intensified	Conventional	Effect estimate (95% CI)	*P* value
8-week epithelialization, n/N (%)	43/52 (82.69)	20/50 (40.00)	RD 42.69% (25.66–59.73); RR 2.07 (1.44–2.97)	<0.001
12-week epithelialization, n/N (%)	46/52 (88.46)	32/50 (64.00)	RD 24.46% (8.57–40.35); RR 1.38 (1.10–1.74)	0.004
KM median time to first recorded epithelialization, weeks	4.00	12.00	Log-rank chi-square = 28.24	<0.001

Binary-outcome *P* values were calculated with Pearson's chi-square test. Risk-difference confidence intervals used the Wald method; risk-ratio confidence intervals were calculated on the log scale. Time to first recorded epithelialization was compared with the log-rank test. Patients without recorded epithelialization by week 12 were administratively censored at 12 weeks. CI, confidence interval; KM, Kaplan–Meier; RD, risk difference; RR, risk ratio.

### 3.3 Eight-week epithelialization and time to first recorded epithelialization

By week 8, recorded epithelialization had been observed in 43/52 intensified-strategy patients and 20/50 conventional-strategy patients (82.69% versus 40.00%; *P* < 0.001; Table 2). The risk difference was 42.69 percentage points (95% CI, 25.66–59.73), and the risk ratio was 2.07 (95% CI, 1.44–2.97). The absolute between-group difference was therefore larger at week 8 than at week 12, while both groups accumulated additional recorded epithelialization events between these assessments.

**Figure 3:**
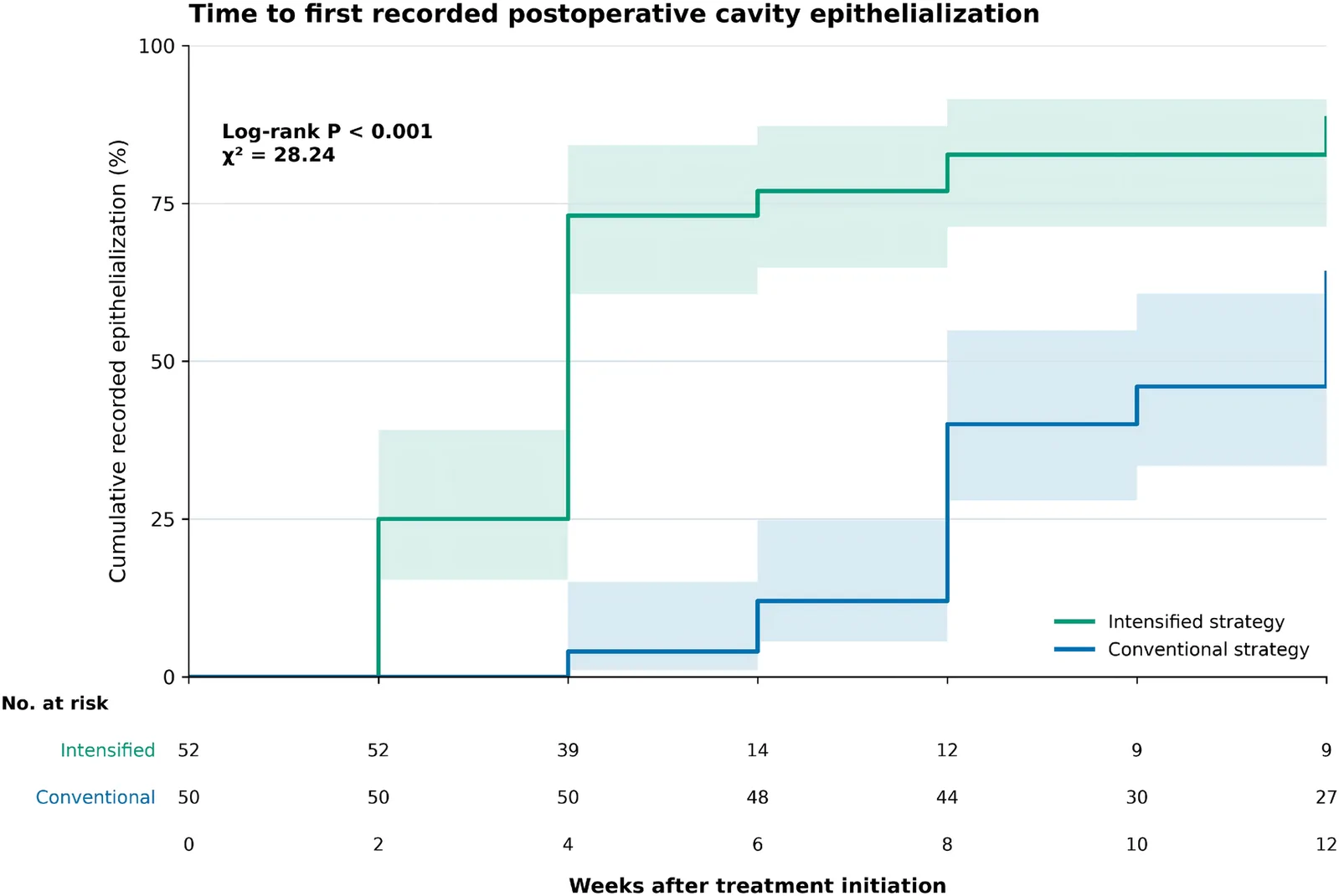
Time to first recorded postoperative cavity epithelialization. Kaplan–Meier complement curves show cumulative recorded epithelialization with 95% confidence bands. Patients without recorded epithelialization by week 12 were administratively censored at 12 weeks. Median time to first recorded epithelialization was 4 weeks in the intensified group and 12 weeks in the conventional group (log-rank *χ*^2^ = 28.24; *P* < 0.001). The event time represents the first recorded epithelialization at an observed visit rather than the exact biological onset.

Kaplan–Meier analysis showed earlier first recorded epithelialization in the intensified group than in the conventional group (log-rank *χ*^2^ = 28.24; *P* < 0.001; Figure 3). The median time to first recorded epithelialization was 4 weeks in the intensified group and 12 weeks in the conventional group. Patients without recorded epithelialization by week 12 were administratively censored at 12 weeks. Because epithelialization was observed only at follow-up visits, these estimates represent the time of first recorded epithelialization rather than the exact biological onset of mucosal repair.

### 3.4 Symptom and endoscopic score improvements

**Figure 4:**
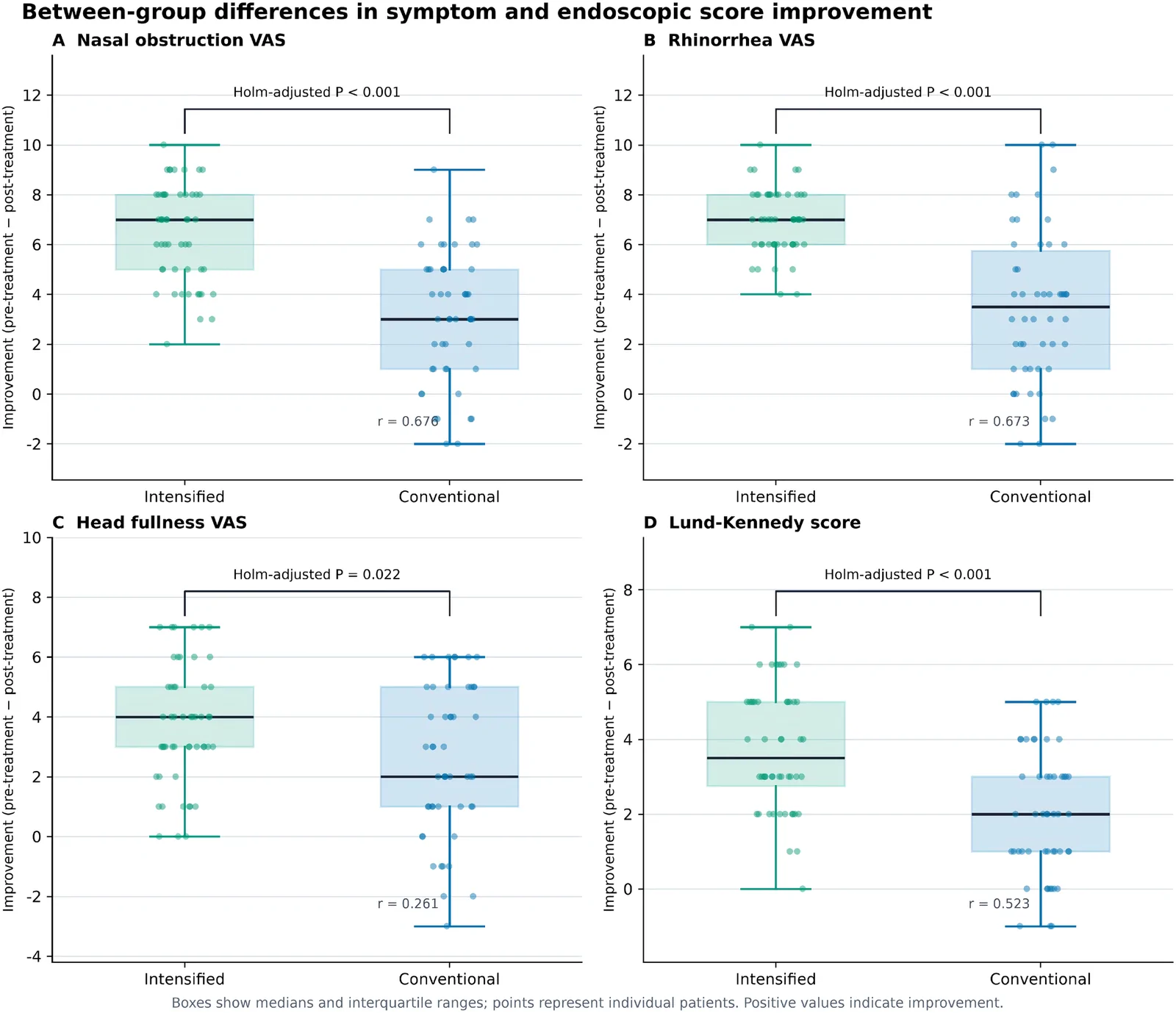
Between-group differences in symptom and endoscopic score improvement. Improvement was defined as pretreatment minus post-treatment score; larger positive values indicate greater improvement. Points represent individual patients, and horizontal bars show medians with interquartile ranges. Brackets report Holm-adjusted two-sided *P* values from Mann–Whitney U tests and rank-biserial effect sizes. The adjusted *P* value for head fullness VAS was 0.022. Group-specific medians, interquartile ranges, and descriptive differences between sample medians are reported in Table 3. VAS, visual analog scale.

Score-improvement distributions were greater in the intensified group for all four secondary outcomes after Holm correction (Table 3; Figure 4). Median nasal obstruction VAS improvement was 7.0 (IQR, 5.0–8.0) versus 3.0 (IQR, 1.0–5.0). The comparison yielded U = 2,179.0, adjusted *P* < 0.001, and rank-biserial r = 0.676. Median rhinorrhea VAS improvement was 7.0 (IQR, 6.0–8.0) versus 3.5 (IQR, 1.0–5.75). The corresponding values were U = 2,175.5, adjusted *P* < 0.001, and r = 0.673.

**Table 3:** Between-group comparisons of symptom and endoscopic score improvement.

Outcome	Intensified improvement	Conventional improvement	U	Holm-adjusted P	Rank-biserial r	Difference between sample medians
Nasal obstruction VAS	7.0 (5.0–8.0)	3.0 (1.0–5.0)	2,179.0	<0.001	0.676	4.0
Rhinorrhea VAS	7.0 (6.0–8.0)	3.5 (1.0–5.75)	2,175.5	<0.001	0.673	3.5
Head fullness VAS	4.0 (3.0–5.0)	2.0 (1.0–5.0)	1,639.0	0.022	0.261	2.0
Lund-Kennedy score	3.5 (2.75–5.0)	2.0 (1.0–3.0)	1,980.0	<0.001	0.523	1.5

Improvement was defined as pretreatment minus post-treatment score and is presented as median (IQR). *P* values are two-sided Mann–Whitney U tests with Holm correction across four outcomes. Positive rank-biserial r values favor the intensified strategy. IQR, interquartile range; VAS, visual analog scale. The descriptive difference is the intensified-strategy sample median minus the conventional-strategy sample median; it is a descriptive contrast and not a Hodges–Lehmann estimate.

Median head fullness VAS improvement was 4.0 (IQR, 3.0–5.0) versus 2.0 (IQR, 1.0–5.0). The corresponding values were U = 1,639.0, adjusted *P* = 0.022, and r = 0.261. Median Lund-Kennedy score improvement was 3.5 (IQR, 2.75–5.0) versus 2.0 (IQR, 1.0–3.0). The comparison yielded U = 1,980.0, adjusted *P* < 0.001, and r = 0.523. Head fullness had the smallest rank-biserial effect size. The descriptive differences between the intensified- and conventional-strategy sample medians were 4.0 points for nasal obstruction VAS, 3.5 points for rhinorrhea VAS, 2.0 points for head fullness VAS, and 1.5 points for the Lund-Kennedy score (Table 3).

### 3.5 Exploratory Firth penalized logistic regression

In the exploratory Firth model, the intensified strategy remained associated with epithelialization recorded by week 12 (adjusted OR, 5.58; 95% CI, 1.87–16.68; *P* = 0.002; Table 4). Age was not independently associated with epithelialization (adjusted OR per year, 0.98; 95% CI, 0.94–1.02; *P* = 0.374). Neither peripheral blood eosinophil percentage nor baseline Lund-Kennedy score was independently associated with epithelialization. The adjusted OR for eosinophil percentage was 1.18 per 1% (95% CI, 0.88–1.59; *P* = 0.277). The adjusted OR for Lund-Kennedy score was 0.71 per point (95% CI, 0.44–1.15; *P* = 0.164). Male sex was not statistically significant and the estimate was imprecise (adjusted OR, 2.90; 95% CI, 1.00–8.40; *P* = 0.0503); it should not be interpreted as evidence of a sex-specific treatment response. The limited number of patients without epithelialization constrained model complexity and precision. Therefore, the adjusted association should be interpreted as exploratory after limited covariate adjustment, rather than as evidence that residual confounding was eliminated.

**Table 4:** Exploratory Firth penalized logistic regression for 12-week epithelialization.

Variable	Adjusted OR	95% CI	*P* value
Intensified vs. conventional strategy	5.58	1.87–16.68	0.002
Age, per year	0.98	0.94–1.02	0.374
Male sex	2.90	1.00–8.40	0.0503
Peripheral blood eosinophils, per 1%	1.18	0.88–1.59	0.277
Baseline Lund-Kennedy score, per point	0.71	0.44–1.15	0.164

The parsimonious model included treatment strategy, age, sex, peripheral blood eosinophil percentage, and baseline Lund-Kennedy score. The outcome was coded 1 for epithelialization recorded by week 12 and 0 otherwise. Values are Firth bias-reduced ORs with Wald 95% CIs. Results are exploratory because the number of patients without recorded epithelialization by week 12 was limited. The unrounded *P* value for male sex was 0.0503; its 95% CI lower bound rounded to 1.00. CI, confidence interval; OR, odds ratio.

### 3.6 Procedural exposure and recorded adverse events

In the intensified group, 39/52 patients (75.0%) underwent at least two debridement sessions, whereas 13/52 (25.0%) underwent one debridement before recorded epithelialization. The median number of debridement sessions per patient was 2 (IQR, 1.75–3.00; range, 1–5). This repeated mechanical exposure was part of the intensified strategy and may itself have influenced short-term cavity patency, endoscopic appearance, and epithelialization recording. Serious adverse events were not documented in the available records of either group during the 12-week observation period. The available records contained no treatment discontinuation attributed to clinically important epistaxis, uncontrolled infection, severe systemic corticosteroid intolerance, or another treatment-related complication. These findings describe retrospectively documented events only and should not be interpreted as evidence of comparative safety. Adverse events were not prospectively solicited, and systematic endocrine, metabolic, ocular, or local safety assessments were not performed. Minor local symptoms, transient discomfort, self-limited bleeding, delayed complications, asymptomatic hypothalamic-pituitary-adrenal axis suppression, ocular pressure changes, or metabolic effects may have been missed or under-recorded in routine clinical notes. Therefore, this study cannot establish safety or exclude uncommon, delayed, asymptomatic, or mild adverse effects.

## 4 Discussion

In this retrospective cohort of adults with postoperative difficult-to-treat CRSwNP, a combined intensified outpatient strategy consisting of targeted in-office endoscopic debridement followed by supine head-hanging budesonide instillation was associated with earlier and more frequent recorded postoperative cavity epithelialization than conventional budesonide nasal spray, on the background of the same short course of oral methylprednisolone. The between-group difference was particularly evident at week 8 and remained present at week 12; time-to-event analysis likewise showed a shorter median time to first recorded epithelialization in the intensified group. Score-improvement distributions for nasal obstruction, rhinorrhea, head fullness, and Lund-Kennedy endoscopic score were also greater in the intensified group after multiplicity correction. No serious adverse events were documented during the 12-week observation period, although safety ascertainment was retrospective and incomplete. Taken together, these findings suggest a consistent association between the combined intensified outpatient strategy and earlier recorded postoperative cavity epithelialization in selected adults with difficult-to-treat CRSwNP.

These findings are compatible with a combined mechanical-pharmacological rationale for postoperative difficult-to-treat CRSwNP, although the present design cannot establish a mechanism or separate component effects. Repeated debridement may reduce obstructive inflammatory tissue and alter short-term endoscopic appearance, whereas positional budesonide delivery changes formulation, nominal dose, contact time, and regional exposure relative to spray therapy. Because these factors were delivered as one package, the between-group difference should be interpreted only as an association with the combined intensified strategy. It cannot be attributed specifically to budesonide instillation, debridement, positioning, dose, or their interaction.

This interpretation is consistent with current concepts of postoperative CRSwNP management. After ESS, the effectiveness of topical corticosteroid therapy depends not only on the anti-inflammatory potency of the drug, but also on whether the medication can reach the operated sinus cavity and remain in contact with the diseased mucosal surface. Previous studies have supported corticosteroid irrigation and other enhanced delivery approaches after ESS, although protocols differ in dose, volume, frequency, device, and head position (12–23). Positional intranasal corticosteroid delivery has been proposed to increase deposition in surgically relevant regions that sprays often fail to reach, including the ethmoid cavity, frontal recess, and olfactory cleft. However, in patients with marked edema, granulation, fibrinous obstruction, or early recurrent polypoid tissue, topical therapy may be most relevant after the obstructive tissue burden has been reduced. The present findings are consistent with, but do not prove, this sequential rationale.

The therapeutic landscape of CRSwNP has expanded substantially with the development of biologics for severe uncontrolled disease with type 2 inflammatory features. In addition to established agents targeting IL-4/IL-13 signaling, IgE, and IL-5 pathways, recent phase 3 studies have further supported upstream epithelial cytokine blockade with tezepelumab and long-acting IL-5 pathway inhibition with depemokimab (25–30). Meta-analytic, registry-based, and comparative real-world studies are also clarifying effectiveness, treatment persistence, switching, systemic corticosteroid burden, and outcome standardization for biologic therapy in CRSwNP (31–35). These advances reinforce the concept of severe CRSwNP as a treatable type 2 inflammatory airway disease. However, postoperative difficult-to-treat CRSwNP is clinically heterogeneous, and early recurrent edema or localized polypoid obstruction does not always mandate immediate biologic therapy or formal revision surgery. In patients with an accessible postoperative cavity in whom the main problem is obstructive inflammatory tissue limiting topical treatment access, a local strategy aimed at restoring sinus-cavity patency and increasing topical corticosteroid exposure may represent a reasonable step-up option. This approach should not be viewed as a substitute for revision surgery in all patients, because severe scarring, fixed anatomic obstruction, middle turbinate lateralization, extensive adhesions, or persistent drainage impairment may still require operative correction according to surgical management principles for CRS (36, 37). Careful patient selection and experienced endoscopic assessment are therefore essential. Within this framework, the intensified outpatient strategy may occupy a practical intermediate position in contemporary postoperative care, before or alongside escalation to biologic therapy or revision surgery when clinically indicated.

The relationship between allergic rhinitis and postoperative difficult-to-treat CRSwNP also warrants consideration. Allergic rhinitis may increase nasal symptom burden and amplify type 2 mucosal inflammation, but CRSwNP should not be viewed as a simple extension of allergic rhinitis. Local IgE production, eosinophilic inflammation, epithelial barrier dysfunction, tissue remodeling, and epithelial-derived cytokines may sustain polypoid inflammation even in patients without classical systemic atopy (9–11). In this study, available inflammatory variables, including serum total IgE and tissue eosinophil counts, were reviewed when available. However, confirmed allergic rhinitis status, allergen sensitization, asthma status and control, anti-allergic treatment, and adherence were not sufficiently standardized for formal effect-modification analysis. Future studies should determine whether confirmed allergic rhinitis, allergen sensitization, or ongoing anti-allergic treatment modifies the response to the combined intensified outpatient strategy. Beyond allergic rhinitis, incomplete documentation of asthma burden, prior ESS burden, previous revision surgery, and prior biologic exposure also limited our ability to determine whether patients with more severe type 2 airway disease, greater surgical history, or previous advanced-therapy exposure had different outcomes under the intensified outpatient strategy.

Treatment burden and implementation requirements are important considerations for any outpatient strategy. In this cohort, serious adverse events were not documented in the available records during the 12-week observation period. The procedure was performed under local anesthesia using familiar endoscopic instruments, and routine sinus-cavity packing was not required. These observations may inform practical implementation in centers with appropriate rhinologic expertise, but they should not be interpreted as evidence of comparative safety. Because safety ascertainment was retrospective, mild local events, delayed complications, asymptomatic hypothalamic-pituitary-adrenal axis suppression, ocular pressure changes, and metabolic effects may have been missed or under-recorded. Future prospective studies should include systematic safety monitoring, including local adverse events, endocrine assessment when higher topical corticosteroid doses are used, ocular pressure evaluation when clinically indicated, metabolic monitoring in at-risk patients, and longer follow-up.

This study has several strengths. Eligibility was re-audited according to the EPOS 2020 difficult-to-treat framework after exclusion of pediatric patients, and all included adults had complete treatment allocation and 12-week recorded postoperative cavity epithelialization data. The analysis reported both absolute and relative effect estimates for binary outcomes, time-to-event results, multiplicity-adjusted comparisons for symptom and endoscopic score improvement, and an exploratory Firth bias-reduced logistic regression model to address sparse non-epithelialization events. Together, these analyses provide a consistent real-world signal that the combined intensified strategy was associated with earlier recorded postoperative cavity epithelialization, while not establishing causality.

Several limitations should also be acknowledged. The retrospective, single-center, nonrandomized design is susceptible to treatment-selection bias and residual confounding, because clinicians may have selected the intensified strategy according to anatomy, inflammatory appearance, prior response, adherence, access, or patient preference. The post-index exclusion of patients who initiated additional potentially confounding treatment during follow-up is a particularly important source of potential selection bias. It may have preferentially removed patients with more severe disease, early nonresponse, or clinical deterioration and may therefore have shifted observed outcomes in both groups in a favorable direction. The resulting cohort is a restricted analytic cohort rather than an all-comers or intention-to-treat population; neither the direction nor the magnitude of this bias can be quantified from the available data. Sequential re-audit showed that 29 of the 48 exclusions occurred because additional potentially confounding treatment was initiated during follow-up. Reporting this mutually exclusive count improves transparency but does not remove the selection bias introduced by the post-index restriction. Future prospective cohorts should enroll patients before treatment allocation, retain participants who require rescue or treatment escalation, and analyze rescue and escalation events as outcomes. Residual confounding also remains possible despite the exploratory Firth model. Important domains were unavailable as standardized analysis variables, including confirmed allergic rhinitis, allergen sensitization, asthma diagnosis and control, prior ESS burden and revision surgery, medication adherence, concomitant anti-allergic or asthma-related treatment, prior biologic exposure, and detailed postoperative inflammatory burden. These factors may have influenced both treatment selection and epithelialization. The adjusted association should therefore be interpreted as exploratory and hypothesis-generating, not as an independent causal effect.

The 12-week observation window captured early recorded postoperative cavity epithelialization only. It was insufficient to evaluate durable disease control, later recurrence, subsequent systemic corticosteroid or antibiotic use, initiation of biologic therapy, revision surgery, or longer-term safety. The findings should therefore not be interpreted as evidence of recurrence prevention or sustained effectiveness. Prospective follow-up of at least 12 months is needed to evaluate recurrence, reoperation, treatment escalation, durability, and delayed adverse events.

Adherence to the prescribed supine head-hanging position and twice-daily instillation schedule was not objectively or systematically recorded. Variation in positioning technique, duration, or frequency may have altered topical exposure and introduced exposure misclassification that could not be incorporated into the adjusted analysis.

A specific limitation concerns the primary endpoint. Recorded postoperative cavity epithelialization was a study-specific, single binary endoscopic outcome derived from a source status field assigned during routine care and has not been widely validated as a standard primary endpoint for CRSwNP disease control. Residual edema or polypoid change, drainage-pathway patency, rescue medication, and subsequent procedures were not separately coded components; the endpoint should not be interpreted as a composite disease-control measure. Assessment relied on routine endoscopy without treatment blinding or independent central adjudication. Retrospective assessors verified whether the source status was supported by contemporaneous documentation and available images, but did not reconstruct a new composite endpoint. Because clinicians knew the treatment received, expectation bias could have influenced whether and when epithelialization was recorded. Although experienced rhinologists harmonized their interpretation and resolved uncertainty by consensus, formal interobserver-agreement statistics were unavailable. The recorded time also reflected the first visit at which epithelialization was documented rather than the exact biological onset, creating timing imprecision. Future prospective studies should use prespecified definitions, blinded image review, formal reliability testing, standardized visit windows, and validated measures such as EPOS disease-control criteria, SNOT-22, objective olfactory testing, and standardized endoscopic scores. These limitations reinforce that the present results show association, not causation.

This study addresses a practical question in postoperative CRSwNP care: how to manage an accessible but inflamed and partially obstructed postoperative cavity when conventional nasal spray delivery may be insufficient. The consistent direction of the epithelialization, time-to-event, symptom, endoscopic, and adjusted analyses supports further evaluation of the combined intensified outpatient strategy. Prospective multicenter component-controlled or factorial studies should enroll patients before treatment allocation, retain patients who require rescue or escalation, use standardized endoscopy windows, blinded image review, validated patient-reported outcomes, objective olfactory testing, prespecified repeat-debridement criteria, and systematic safety assessment, and continue follow-up for at least 12 months to evaluate durability and the independent contribution of each treatment component.

## 5 Conclusion

In adults with postoperative difficult-to-treat CRSwNP, a combined intensified outpatient strategy consisting of targeted in-office endoscopic debridement followed by supine head-hanging budesonide instillation was associated with earlier and more frequent recorded postoperative cavity epithelialization than conventional budesonide nasal spray, on the background of the same short systemic corticosteroid course. The strategy may represent a local step-up option for selected patients with accessible postoperative cavities and obstructive inflammatory tissue, but it should not be viewed as a substitute for biologic therapy or revision surgery when established indications are present. The present data do not establish the independent effect of any single component, prove causality, or demonstrate comparative safety. Prospective multicenter, component-controlled or factorial studies with follow-up of at least 12 months, systematic safety assessment, and retention of patients requiring rescue or escalation are needed to test causal relationships, evaluate durability, refine patient selection, and determine the independent contribution of each treatment element. Because follow-up ended at 12 weeks, these findings do not demonstrate long-term disease control, recurrence prevention, avoidance of biologic escalation, or avoidance of revision surgery.

## Data Availability

The raw data supporting the conclusions of this article will be made available by the authors, without undue reservation.
