# Supplementary material for: Combined in-office endoscopic debridement and supine head-hanging budesonide instillation for postoperative difficult-to-treat chronic rhinosinusitis with nasal polyps: a retrospective cohort study
Source: Front Allergy. 2026 Aug 25;7:1914838. doi: 10.3389/falgy.2026.1914838 (PMC13549901; doi:10.3389/falgy.2026.1914838)
Supplement: Supplementary file 1 [file Table1.docx]

**Supplementary Table S1. Screening flow and reasons for exclusion from the final analytic cohort**

| **Screening step / exclusion reason** | **Excluded, n** | **Remaining, n** |
| --- | --- | --- |
| Initial screened postoperative CRSwNP records reassessed against EPOS 2020 difficult-to-treat criteria | — | 150 |
| Excluded: age <18 years | 3 | 147 |
| Excluded: inability to safely adopt the supine head-hanging position | 2 | 145 |
| Excluded: severe cardiovascular or cerebrovascular disease | 1 | 144 |
| Excluded: uncontrolled hypertension or diabetes | 2 | 142 |
| Excluded: active peptic ulcer disease | 1 | 141 |
| Excluded: severe osteoporosis or glaucoma | 1 | 140 |
| Excluded: severe hepatic, renal, or hematologic disease | 2 | 138 |
| Excluded: hypersensitivity to study medications | 1 | 137 |
| Excluded: cystic fibrosis, primary ciliary dyskinesia, or fungal sinusitis | 2 | 135 |
| Excluded: pregnancy or lactation | 1 | 134 |
| Excluded: incomplete records or indeterminate 12-week endpoint | 3 | 131 |
| Excluded: initiation of additional confounding treatments during the 12-week observation period | 29 | 102 |
| **Total excluded** | **48** | **102** |
| **Final analytic cohort** | **—** | **102** |
| Final cohort: combined intensified outpatient strategy | — | 52 |
| Final cohort: conventional strategy | — | 50 |

Note: During re-audit, exclusion criteria were applied sequentially in the hierarchical order shown. Each record was counted only once under the first criterion met in this sequence; therefore, reason-specific exclusion counts were mutually exclusive and summed to 48. ‘Remaining, n’ indicates the number of records remaining after sequential application of each exclusion criterion. Additional potentially confounding treatments included biologics, antihistamines, leukotriene receptor antagonists, antibiotics, allergen immunotherapy, or additional systemic corticosteroids initiated during the 12-week observation period. CRSwNP, chronic rhinosinusitis with nasal polyps; EPOS, European Position Paper on Rhinosinusitis and Nasal Polyps.
